# Targeting the ISG15+STAT1+ monocyte-driven inflammatory storm with Fedratinib in traumatic lung injury via the JAK2/STAT3/PIM1 axis

**DOI:** 10.3389/fimmu.2026.1843701

**Published:** 2026-06-19

**Authors:** Kun Zhang, Dan Li, Le Gao, Mingwei Chen

**Affiliations:** Department of Respiratory and Critical Care Medicine, First Affiliated Hospital of Xi’an Jiaotong University, Xi’an, Shaanxi, China

**Keywords:** Fedratinib, monocyte heterogeneity, PIM1, single-cell RNA sequencing, traumatic lung injury

## Abstract

**Background:**

Traumatic lung injury (TLI) frequently progresses to acute respiratory distress syndrome (ARDS), a condition with high mortality and limited targeted therapies. This study aimed to identify specific immune cell drivers and potential therapeutic targets for precision intervention in TLI.

**Methods:**

We conducted a comprehensive analysis of single-cell RNA sequencing (scRNA-seq) data from peripheral blood mononuclear cells of trauma patients, transcriptomic data from alveolar macrophages in ARDS patients, and lung tissue transcriptomic data from a TLI mouse model. An integrated bioinformatics approach was employed, including the use of the Augur and Milo algorithms for prioritizing cellular perturbations, Monocle 3 for trajectory inference, and two-sample Mendelian randomization (TSMR) for causal inference. Potential drugs were screened using Connectivity Map, target-specificity predictions were made using the scRANK algorithm, and validation was performed using an *in vitro* macrophage polarization model.

**Results:**

The scRNA-seq analyses revealed that classical monocytes showed the greatest disturbance and response following trauma. Among these, the ISG15+STAT1+ monocyte subpopulation expanded post-trauma and drove an inflammatory storm through robust M1-like polarization. PIM1 was identified as a core pathogenic gene co-upregulated in both peripheral blood and lung tissue. Further TSMR analysis confirmed elevated PIM1 expression as a causal risk factor for ARDS. Concurrently, the JAK2/STAT3/PIM1 pathway is activated synchronously during the acute trauma phase. Drug repurposing research suggests that the JAK2 inhibitor Fedratinib can reverse the transcriptomic characteristics of TLI. *In vitro* experiments have demonstrated that Fedratinib effectively inhibits M1 polarization and the expression of inflammatory genes by interfering with the JAK2/STAT3/PIM1 signaling axis.

**Conclusions:**

This study identifies the JAK2/STAT3/PIM1 signaling axis within the ISG15+STAT1+ monocyte subpopulation as a key driver of TLI and a potential therapeutic target, and suggests that fedratinib is a potential candidate for the targeted treatment of TLI.

## 1 Introduction

Injuries, including trauma, are the leading causes of death and disability for individuals under the age of 45 worldwide (1–3). Among various types of traumas, chest traumas account for 10%–15% of all trauma admissions and contribute to approximately 25% of trauma-related deaths (4, 5). Traumatic lung injury (TLI) generally refers to the destruction of lung parenchyma caused by external forces acting directly on the chest. According to the Western Trauma Association, TLI is a distinct clinic-pathological entity characterized primarily by the mechanical rupture of the alveolar-capillary barrier, followed by hemorrhage and edema (6). TLI presents as direct anatomical damage to the impacted area. This mechanical trauma acts as the initiating factor for a subsequent cascade of pathophysiological reactions (7, 8). Once trauma patients develop acute respiratory distress syndrome (ARDS), the mortality rate can reach as high as 56%, significantly exceeding the 13% observed in patients without ARDS (9). The progression of TLI follows the classic paradigm of “mechanical impact initiation followed by immune recognition/inflammatory storm.” This “injury first, secondary inflammation” model fundamentally distinguishes TLI from pathogen-driven infectious ARDS (10, 11).

Whether ARDS patients eventually achieve pulmonary functional recovery or suffer irreversible lung fibrotic damage depends directly on the spatiotemporal dynamic balance of alveolar macrophage polarization states across different stages of the disease (12). During the initial phase of ARDS onset, M1-polarized macrophages trigger a “cytokine storm,” releasing inflammatory mediators such as TNF-α, IL-1β, and IL-6, which further damage lung tissue (13, 14). The “polarization block” from M1 to M2 is a core mechanism underlying poor prognosis and mortality in ARDS (15).

The JAK/STAT pathway is one of the central signaling axes regulating macrophage polarization (16). PIM1 expression is maintained at extremely low levels in resting cells but can be rapidly and potently induced by various external signals, including cytokines. The core signaling pathway mediating this process is the JAK/STAT pathway (17). This positioning makes PIM1 a critical early effector gene in inflammatory and immune responses, capable of rapidly converting external inflammatory or growth signals into intracellular functional responses.

However, bulk transcriptomic analysis often treats cell populations as homogeneous. This “averaging effect” masks the high degree of heterogeneity in the transcriptomic profiles and functional states of myeloid cells, making it difficult to precisely pinpoint the core pathogenic subclusters and molecular switches driving the inflammatory storm in TLI. To overcome this limitation, it is necessary to examine the immune microenvironment at a resolution that matches the complexity of the disease. This study aims to utilize single-cell RNA sequencing (scRNA-seq) and multi-omics integration analysis, using trauma as the entry point, to map the dynamic scRNA-seq landscape of peripheral blood mononuclear cells (PBMCs). By further deconstructing monocyte heterogeneity, we seek to reveal the specific transcriptional signatures and key regulatory networks of these subclusters and map them to ARDS. This approach aims to elucidate the molecular mechanisms underlying immune system pathology and to identify potential therapeutic targets for precision immune intervention in ARDS.

## 2 Materials and methods

### 2.1 Data sources

This study integrated multiple public databases to obtain bulk transcriptomics, scRNA-seq, and Genome-Wide Association Study (GWAS) summary data. Bulk transcriptomic datasets were sourced from the GEO database, including the alveolar macrophage dataset GSE116560 from patients with ARDS, the longitudinal peripheral blood leukocyte expression profile GSE36809 from patients with severe trauma, and the lung tissue transcriptomic dataset GSE172209 from a mouse model of TLI. The raw scRNA-seq data PRJNA683058 encompass PBMC samples from trauma patients collected at 4 h, 24 h, and 72 h post-trauma. Additionally, GWAS summary statistics for ARDS were obtained from the FinnGen database (R10 release), and cis-expression quantitative trait loci (eQTL) data were retrieved from the eQTLGen consortium.

### 2.2 The scRNA-seq analysis

Raw scRNA-seq data were aligned to the human reference genome GRCh38 using Cell Ranger software, followed by aggregation and normalization. Quality control was performed using the Seurat R package (v5.1.0), filtering for high-quality cells with detected gene counts between 200 and 6,000 and a mitochondrial gene proportion of less than 15%. To integrate samples and account for technical variability, the Harmony algorithm was employed to remove batch effects. Based on dimensionality reduction and clustering analysis, 14 major immune cell lineages were identified using the ScType R package and canonical marker genes. Subsequently, the Milo algorithm was applied for differential abundance testing to identify cell neighborhoods significantly enriched under traumatic stress. To quantify the transcriptional sensitivity of each subpopulation to traumatic perturbation, the Augur algorithm was used to calculate area under the curve (AUC) scores, thereby prioritizing cellular responses.

### 2.3 Fine deconstruction of myeloid cells, polarization assessment, and trajectory analysis

To resolve heterogeneity in myeloid cells, independent re-clustering was performed on monocyte and dendritic cell subpopulations (18–23). Using the AddModuleScore algorithm from the Seurat package, functional polarization scores for monocyte subpopulation were calculated based on a classic gene set: the M1-like pro-inflammatory score (M1_Score: IL1B, TNF, IL6, CXCL10/11, NOS2, CD80, CD86, IRF5) and the M2-like anti-inflammatory/repair score (M2_Score: ARG1, MRC1, CD163, PPARG, IRF4, CCL24, CLEC4A), and incorporating the results into the metadata. Furthermore, Nebulosa density plots were utilized to visualize the expression abundance of key pro-inflammatory factors at single-cell resolution. Developmental trajectory inference was conducted using the Monocle 3 algorithm, designating homeostatic monocyte subpopulations as the starting point to resolve the evolutionary path of monocytes toward pathogenic branches. Additionally, the decoupleR R package was used to infer transcription factor activity within each subcluster. The irGSEA R package was combined with the Metascape platform to perform biological functional enrichment and molecular interaction network analysis on core differentially expressed genes (DEGs). During the differential expression analysis, in order to retain as many potential differential signals as possible, the minimum expression percentage (min.pct) for a gene in any cell cluster was set to 0.1, the |log_2_(Fold change)| threshold (avg_log_2_FC) to 0.8, and the adjusted *P*-value (*P*_val_adj) to <0.05.

### 2.4 Causal inference and temporal expression clustering

By comparing the alveolar macrophage transcriptomes of ARDS patients with different prognoses, this study identified core pathogenic targets co-upregulated in both PBMC pathogenic subclusters and locally damaged tissues. The criteria for identifying DEGs were set as *P* < 0.05 and |Fold Change| > 1.2, using the limma R package. For the candidate gene PIM1, two-sample Mendelian Randomization (TSMR) was employed to evaluate its causal effect on ARDS risk. Causal effects were estimated using the Inverse Variance Weighted method. Furthermore, the Mfuzz algorithm was used to identify the temporal expression patterns of leukocyte genes in severe trauma patients, with a specific focus on clusters involving JAK2, STAT3, and PIM1 to validate the temporal synergy of this signaling axis. For mouse TLI transcriptomic data, cross-species conserved pathogenic signaling pathways were identified through differential expression analysis and human-mouse orthologous gene intersection. Differential expression analysis of TLI mouse lung tissue using DESeq2 (screening criteria: *P*_(adj) < 0.05, |log_2_FC| > 0.5). Enrichment analysis of DEGs using the Metascape website and the gseapy package.

### 2.5 Potential drug repurposing prediction and single-cell sensitivity evaluation

To identify potential drugs capable of reversing the pathological features of TLI, small-molecule drug screening was conducted using the Connectivity Map (CMap) platform. Genes significantly up-regulated in the TLI transcriptome of the above-mentioned mice were used as query features for comparison with drug expression profiles based on the CMap database. The results were quantified using a Tau score (–100 to +100): a negative score (close to –100) indicates a negative correlation with the expression profile, suggesting the ability to reverse TLI-induced changes; a positive score (close to +100) indicates pathological mimicry. This analysis identified Fedratinb as a potential therapeutic agent and validated its therapeutic potential. Subsequently, the scRANK algorithm quantified the drug response sensitivity ranking of different myeloid subclusters to the JAK2 inhibitor across various time points, and projected it onto single-cell dimensionality reduction maps to visualize the distribution of sensitivity. The scRANK algorithm uses default parameters.

### 2.6 *In vitro* experiments

For *in vitro* validation, we used the RAW264.7 macrophage cell line, and established an M1 polarization model induced by IFN-γ and LPS. The RAW264.7 cell line used in this study was obtained from Procell Life Science & Technology Co., Ltd. (Wuhan, China). The CCK-8 assay was used to determine the effect of Fedratinib on cell viability and to calculate the IC50 value. For pharmacological inhibition experiments, polarized cells were treated with varying concentrations of Fedratinib(0.5, 1, and 2 μM). Flow cytometry was used to determine the proportion of CD86+CD206- cells to assess the drug’s effect on M1 phenotypic switching. At the molecular level, RT-qPCR was used to measure mRNA levels of inflammatory genes (*Il1b*, *Il6*, and *Tnf*) and *Pim1*. The primer sequences are shown in **Supplementary Table 1**. Western Blot was performed to detect site-specific phosphorylation of JAK2 and STAT3, along with the protein expression of PIM1, iNOS, and CD86, to systematically evaluate the inhibitory effect of Fedratinib on signaling axis activation. Detailed experimental methods are provided in the Supplementary Methods.

### 2.7 Statistical analysis

Quantitative data are expressed as mean ± standard deviation (SD) and are derived from at least three independent biological replicates. Statistical analysis was performed using one-way analysis of variance (ANOVA), followed by Dunnett’s *post hoc* test for multiple comparisons. All analyses and curve fitting were performed using GraphPad Prism 9.0 software (GraphPad Software, San Diego, CA, USA). Significance was defined as: *P < 0.05, **P < 0.01, ***P < 0.001, ****P < 0.0001.

## 3 Results

### 3.1 Single-cell resolution landscape of the trauma-induced peripheral immune microenvironment

**Figure 1:**
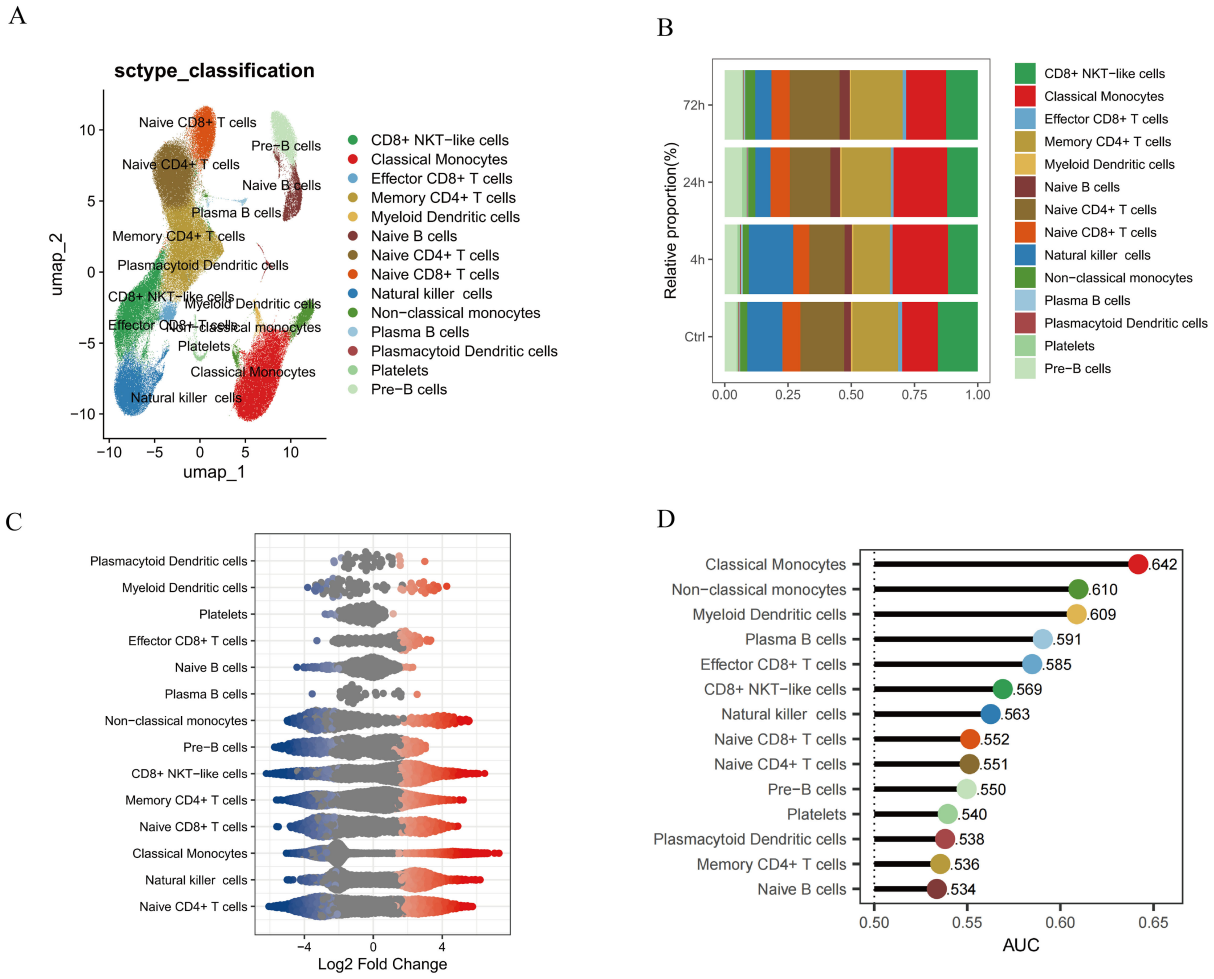
Human PBMC single-cell landscape and response prioritization after severe trauma. **(A)** UMAP plot of 14 core immune cells. **(B)** Stacked bar chart showing temporal composition shifts (Ctrl, 4h, 24h, and 72h). **(C)** Milo plot showing enriched cell neighborhoods under traumatic stress. **(D)** Augur plot showing the cell subsets most sensitive to biological disturbance.

To resolve the systemic immune remodeling following severe trauma, we analyzed the PBMC scRNA-seq from trauma patients (at 4h, 24h, and 72h post-trauma) and healthy controls. Based on dimensionality reduction and clustering analysis, 14 major immune cell lineages were successfully identified (**Figure 1A**). Proportional statistics revealed a significant “myeloid bias” in peripheral blood post-trauma, characterized by an explosive expansion of classical monocyte populations (**Figure 1B**).

Using differential abundance analysis (Milo), we found that specific cellular neighborhoods within the monocyte lineage exhibited significant topological enrichment under traumatic stress (**Figure 1C**). Subsequently, the Augur algorithm was applied to quantify the transcriptomic response intensity of each subpopulation to traumatic stimulation. The results showed that classical monocytes had the highest prioritization score, with an AUC of 0.642 (**Figure 1D**), establishing them as the core effector cells of the trauma-induced acute inflammatory response.

### 3.2 Transcriptomic reprogramming and pro-inflammatory regulatory hubs of classical monocytes

**Figure 2:**
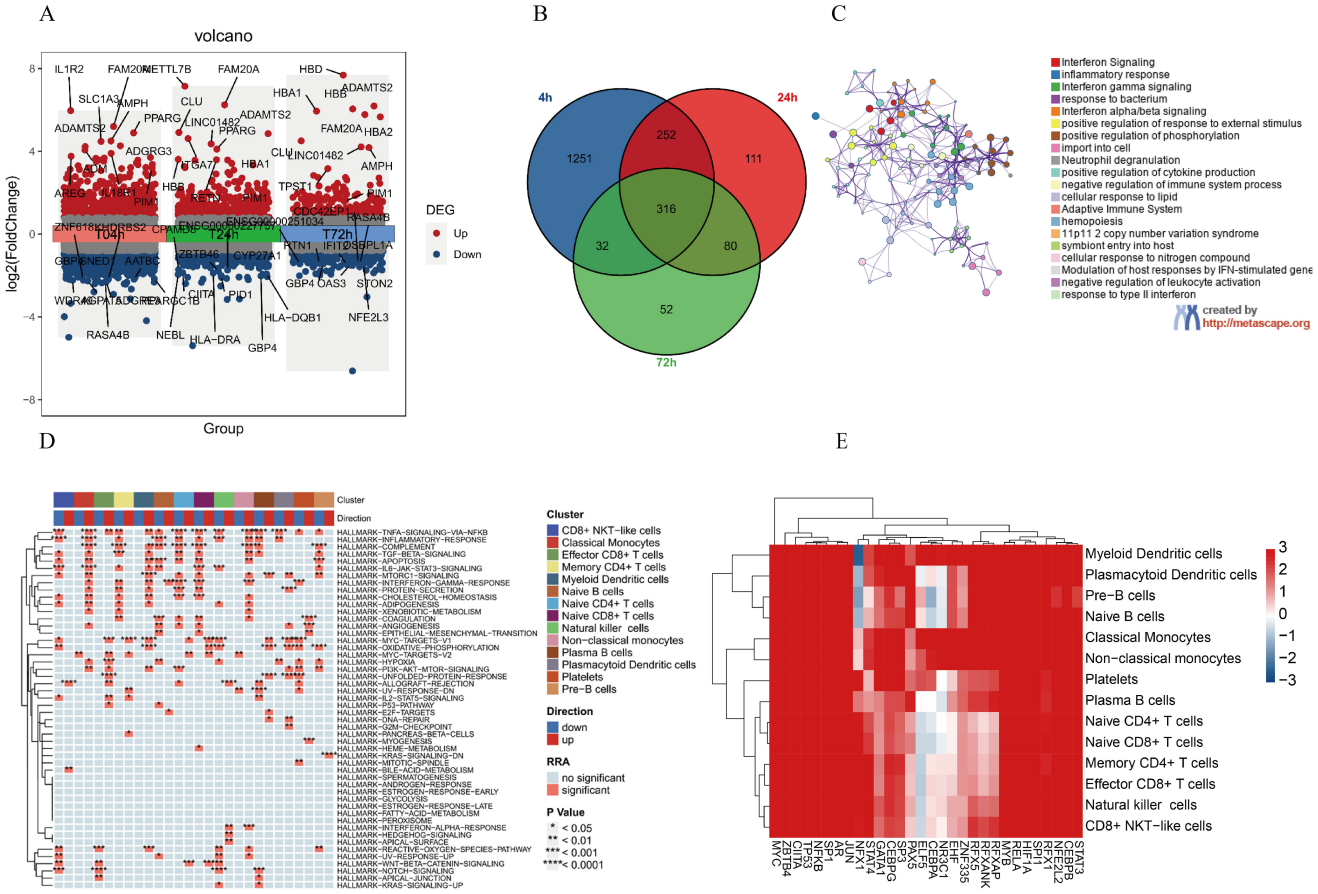
Temporal transcriptomic profiles and regulatory hubs of classical monocytes. **(A)** Volcano plots of DEGs at 4h, 24h, and 72h post-trauma. **(B)** Venn diagram showing the intersection of DEGs identifying core response genes. **(C)** Metascape analysis of core gene functional enrichment and molecular networks. **(D)** irGSEA heatmap of MSigDB Hallmark pathway activity across immune cell subsets. **(E)** decoupleR heatmap displaying inferred transcription factor activity.

Focusing on the most significantly perturbed classical monocytes, we identified 316 core response genes that were consistently differentially expressed across time points by comparing the post-injury stages with the control group (**Figures 2A, B**; **Supplementary Tables 2**–**S4**). Metascape functional enrichment analysis revealed that these genes are highly clustered in key biological processes, including interferon signaling, inflammatory response, and interferon gamma signaling (**Figure 2C**; **Supplementary Table 5**).

The irGSEA analysis showed that core pro-inflammatory pathways (such as the IL6-JAK-STAT3 signaling axis and TNF-alpha signaling via NF-kappaB) exhibited explosive “pulse” activation post-trauma, and this immune stress state did not fully subside within 72 h (**Figure 2D**). Transcription factor activity analysis inferred by the decoupleR algorithm further identified STAT3 and RELA as the core molecular switches driving monocyte transcriptomic reprogramming (**Figure 2E**).

### 3.3 Identification and trajectory analysis of the pathogenic cMo_ISG15+_STAT1+ subcluster

**Figure 3:**
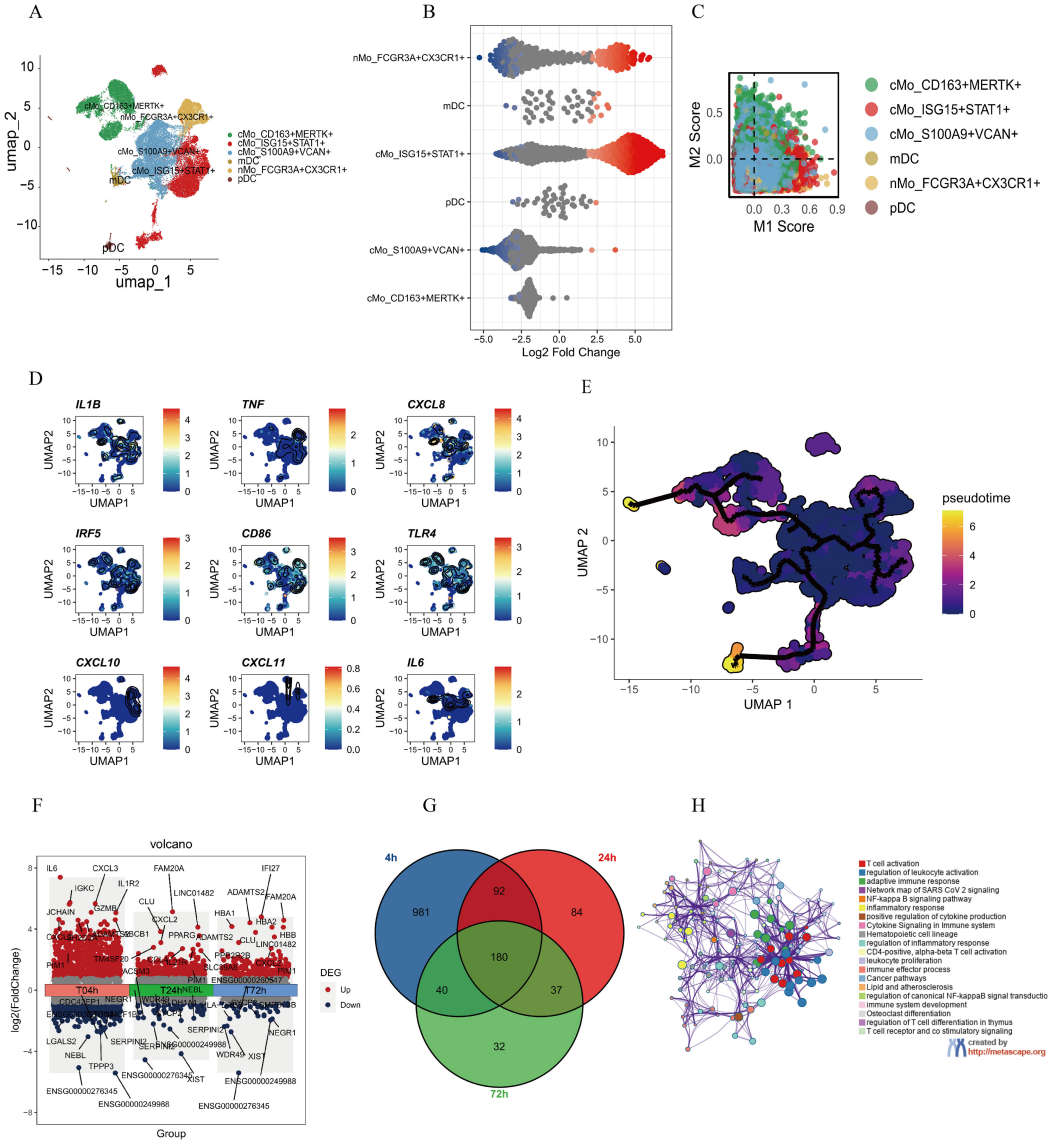
Identification and differentiation trajectory of the pathogenic cMo_ISG15+STAT1+ cell subcluster. **(A)** Re-clustered UMAP of myeloid cells. **(B)** Milo plot showing enriched myeloid cell neighborhoods under trauma. **(C)** Scatter plot of M1/M2 polarization scores. **(D)** Nebulosa density plots of pro-inflammatory markers. **(E)** Monocle 3 trajectory showing the transition from homeostatic to pathogenic states. **(F)** Volcano plot and **(G)** Venn diagram showing DEGs and intersecting DEGs in pathogenic cell subclusters. **(H)** Metascape enrichment analysis of core genes driving the inflammatory response.

Re-clustering of myeloid cells identified a characteristic pathogenic subcluster that emerged early post-trauma, designated as the cMo_ISG15+STAT1+ monocyte subcluster (**Figure 3A**), which is significantly disturbed by trauma (**Figure 3B**). Functional scoring indicated that this subcluster exhibited an extremely high M1-like pro-inflammatory score and an exceptionally low M2-like reparative score (**Figure 3C**). Visualization using Nebulosa density plots revealed that core mediators of the inflammatory storm, such as IL1B, TNF, and IL6, were highly concentrated in the cMo_ISG15+STAT1+ monocyte subcluster (**Figure 3D**).

Monocle 3 pseudotime trajectory inference revealed a clear lineage bifurcation: homeostatic cMo_S100A9+VCAN+ subclusters rapidly evolved toward the cMo_ISG15+STAT1+ pro-inflammatory branch upon traumatic stimulation (**Figure 3E**). Longitudinal differential expression analysis of this cell subset revealed sustained upregulation of inflammatory genes such as PIM1 (**Figure 3F**; **Supplementary Tables 6**–**S8**). By intersecting DEGs from different time points, 180 core pathogenic genes were identified (**Figure 3G**). These genes are highly enriched in pathways such as the “NF-kappa B signaling pathway” and “Cytokine Signaling in the Immune System”, constituting the molecular hub for sustained inflammatory output (**Figure 3H**; **Supplementary Table 9**).

### 3.4 Cross-tissue consistency and genetic causal evidence of PIM1-driven lung injury

**Figure 4:**
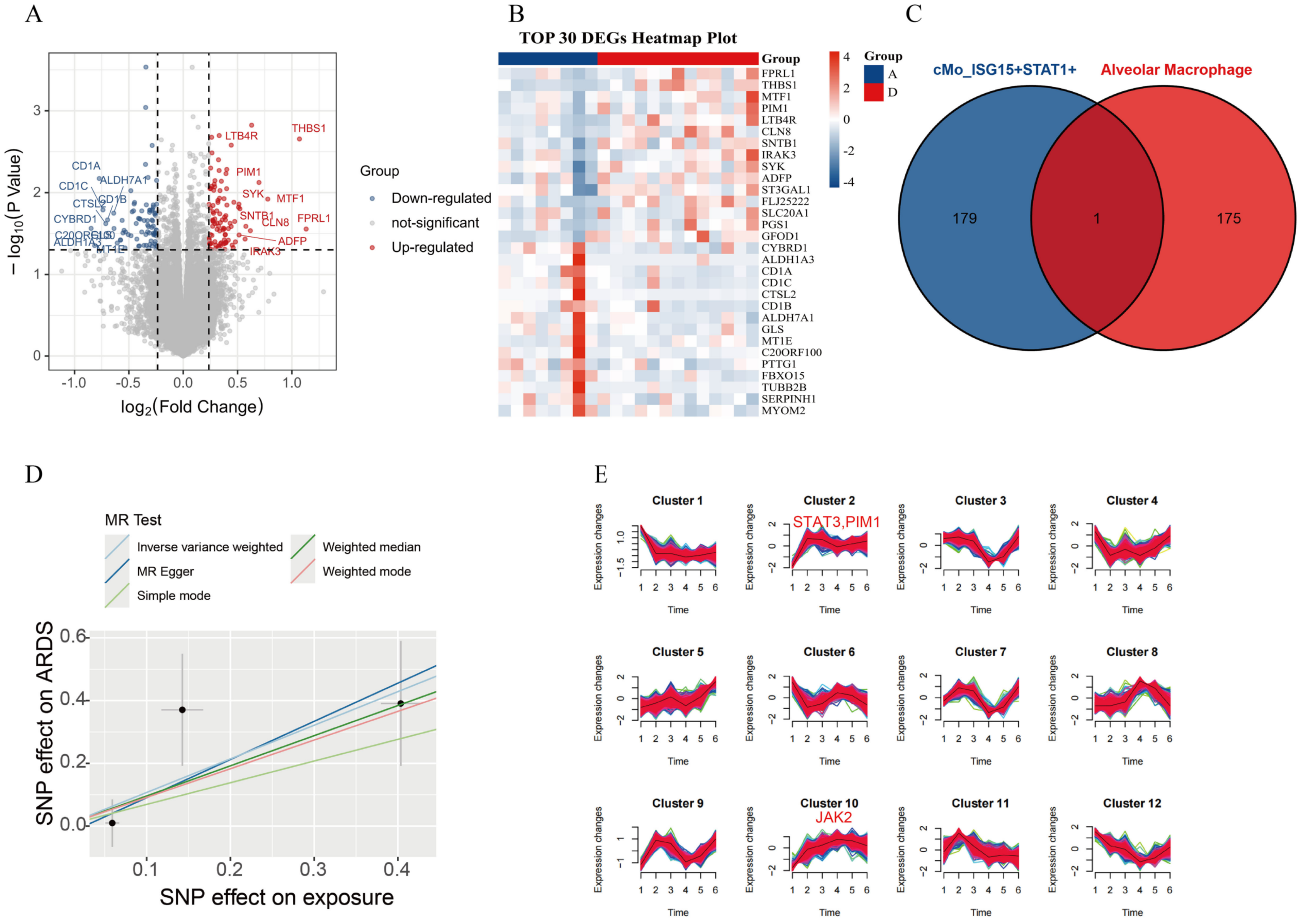
Shared PIM1 characteristics across tissues and genetic causal validation. **(A, B)** Volcano plot and heatmap of ARDS alveolar macrophage DEGs. **(C)** Venn diagram showing the intersection of peripheral pathogenic monocyte genes with lung DEGs to identify conserved targets. **(D)** TSMR scatter plot establishing the causal link between elevated PIM1 expression and ARDS risk. **(E)** Mfuzz analysis of the temporal expression patterns of leukocyte genes in severe trauma patients.

To validate the clinical translational value of our findings, this study compared the alveolar macrophage transcriptomes of ARDS patients with different prognoses. We found that PIM1 expression was significantly elevated in alveolar macrophages from patients with ARDS with poor prognosis (**Figure 4A, B**). Furthermore, PIM1 was the only gene up-regulated in both cMo_ISG15+STAT1+ monocytes in peripheral blood and alveolar macrophages of trauma patients (**Figure 4C**).

TSMR analysis indicated that elevated PIM1 gene expression is significantly associated with ARDS, supporting a potential causal contribution of PIM1 to ARDS susceptibility (**Figure 4D**; **Supplementary Tables 10**, **S11**). The Mfuzz analysis of an independent trauma cohort further showed that JAK2, STAT3, and PIM1 exhibited highly synchronized expression trajectories during the acute phase of trauma, confirming the synergistic activation pattern of this signaling axis *in vivo* (**Figure 4E**).

### 3.5 Cross-species validation and tissue immune microenvironment analysis in TLI mouse models

**Figure 5:**
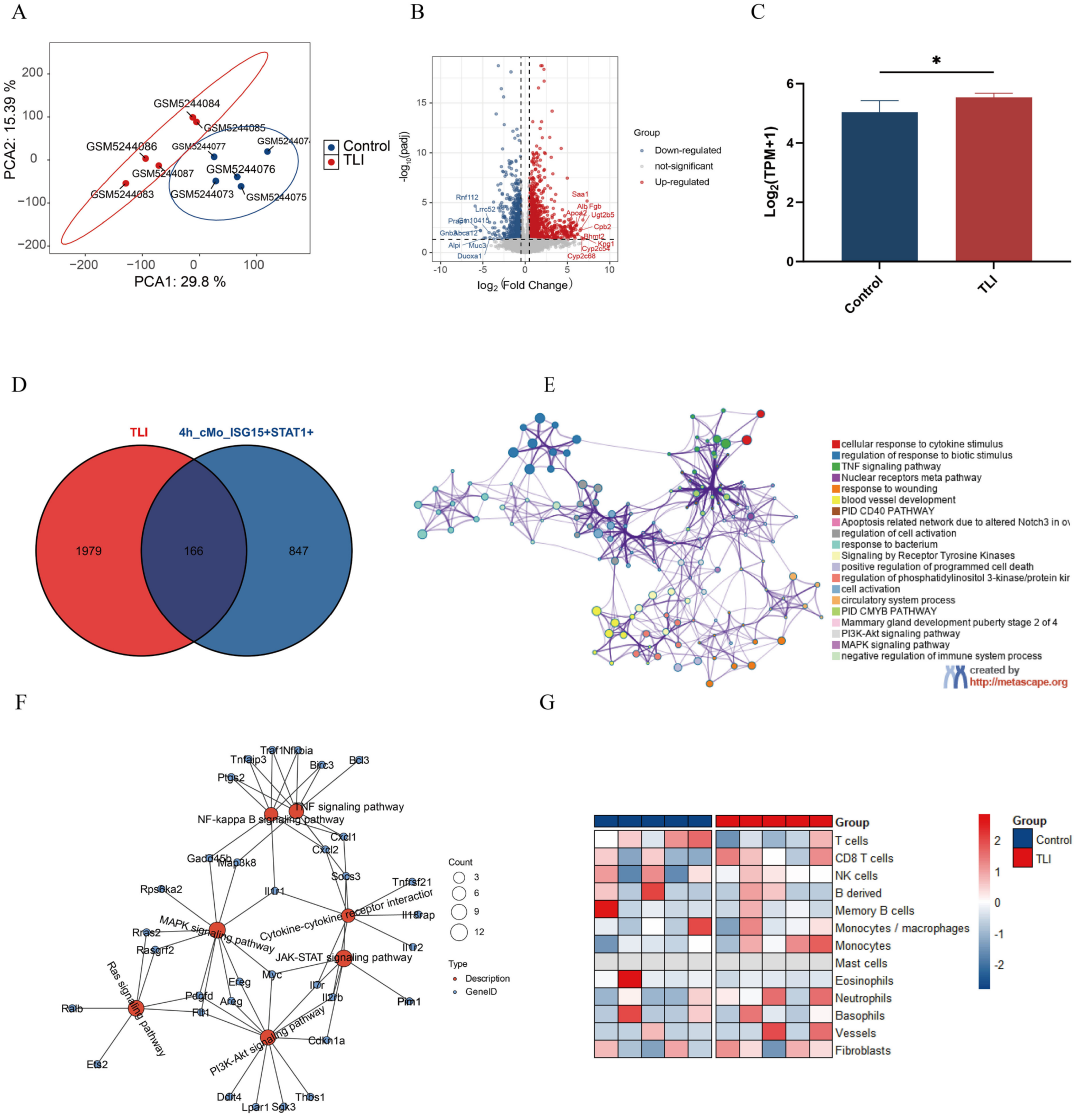
Cross-species validation of TLI characteristics and lung microenvironment. **(A–C)** PCA plot, volcano plot, and bar chart showing significant Pim1 upregulation in mouse TLI lung tissue. **(D)** Venn diagram identifying conserved pathogenic genes between humans and mice. **(E, F)** Functional enrichment and Gene-Concept Network of intersecting genes highlighting key conserved pathways. **(G)** Immune cell infiltration in TLI mouse lung tissue.

In the mouse TLI model, principal component analysis and differential expression analysis confirmed that chest impact induced a drastic remodeling of the lung tissue gene expression profile (**Figures 5A, B**), with the core pathogenic gene Pim1 significantly overexpressed (**Figure 5C**). Human-mouse data intersection revealed 166 conserved pathogenic genes (**Figure 5D**), which were highly enriched in the “cellular response to cytokine stimulus”, “TNF signaling pathway”, and “response to wounding” pathways (**Figure 5E**; **Supplementary Table 12**). KEGG enrichment analysis using the gseapy package revealed that these genes are highly enriched in the JAK-STAT and NF-κB signaling pathways (**Figure 5F**). Immune infiltration analysis confirmed a deep myeloid-dominant infiltration pattern in damaged lung tissue, with significantly increased abundance scores for monocytes and macrophages, highly consistent with the cellular dynamics observed in human peripheral blood (**Figure 5G**).

### 3.6 Precision treatment value of Fedratinib based on drug repurposing prediction

**Figure 6:**
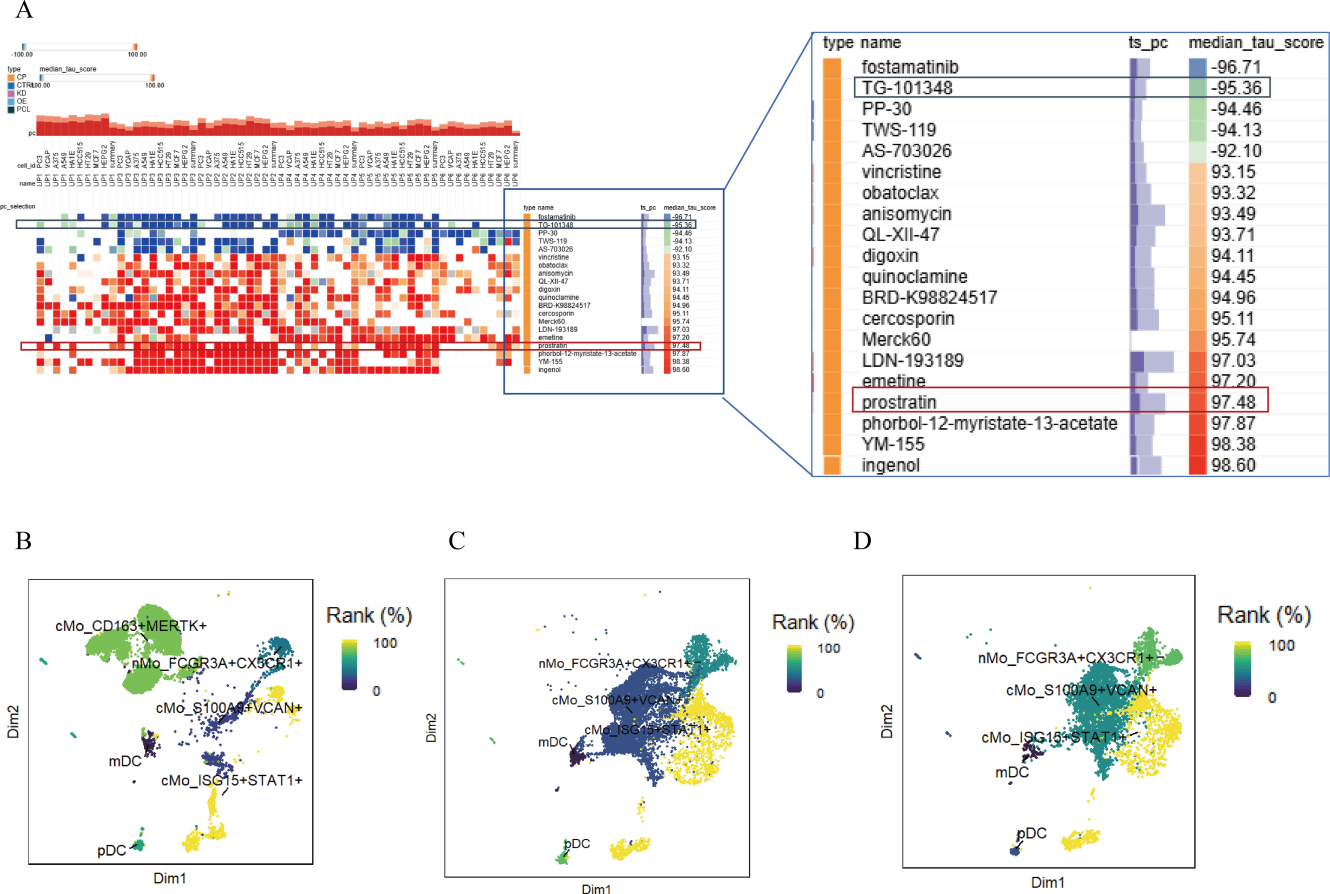
Screening and single-cell validation of therapeutic candidates. **(A)** CMap screening displaying drug perturbation profiles and ranking by Tau score. **(B–D)** scRANK UMAPs showing predicted Fedratinib sensitivity across cell subclusters at 4h **(B)**, 24h **(C)**, and 72h **(D)**.

Based on the transcriptomic signatures of the mouse TLI, CMap analysis screened the selective JAK2 inhibitor Fedratinib (TG-101348), which yielded a median Tau score of -95.36, indicating a potent potential to reverse pathological features. In contrast, the NF-κB activator prostratin was able to mimic the pathological transcriptional profile of TLI (**Figure 6A**; **Supplementary Table 13**). The scRANK simulation predicted that the cMo_ISG15+STAT1+ subcluster exhibited extremely high drug sensitivity to Fedratinib at multiple time points post-trauma, and this sensitivity remained stable from 4 to 72 h post-trauma (**Figures 6B–D**).

### 3.7 *In vitro* validation: Fedratinib inhibits M1 polarization by blocking the signaling axis

**Figure 7:**
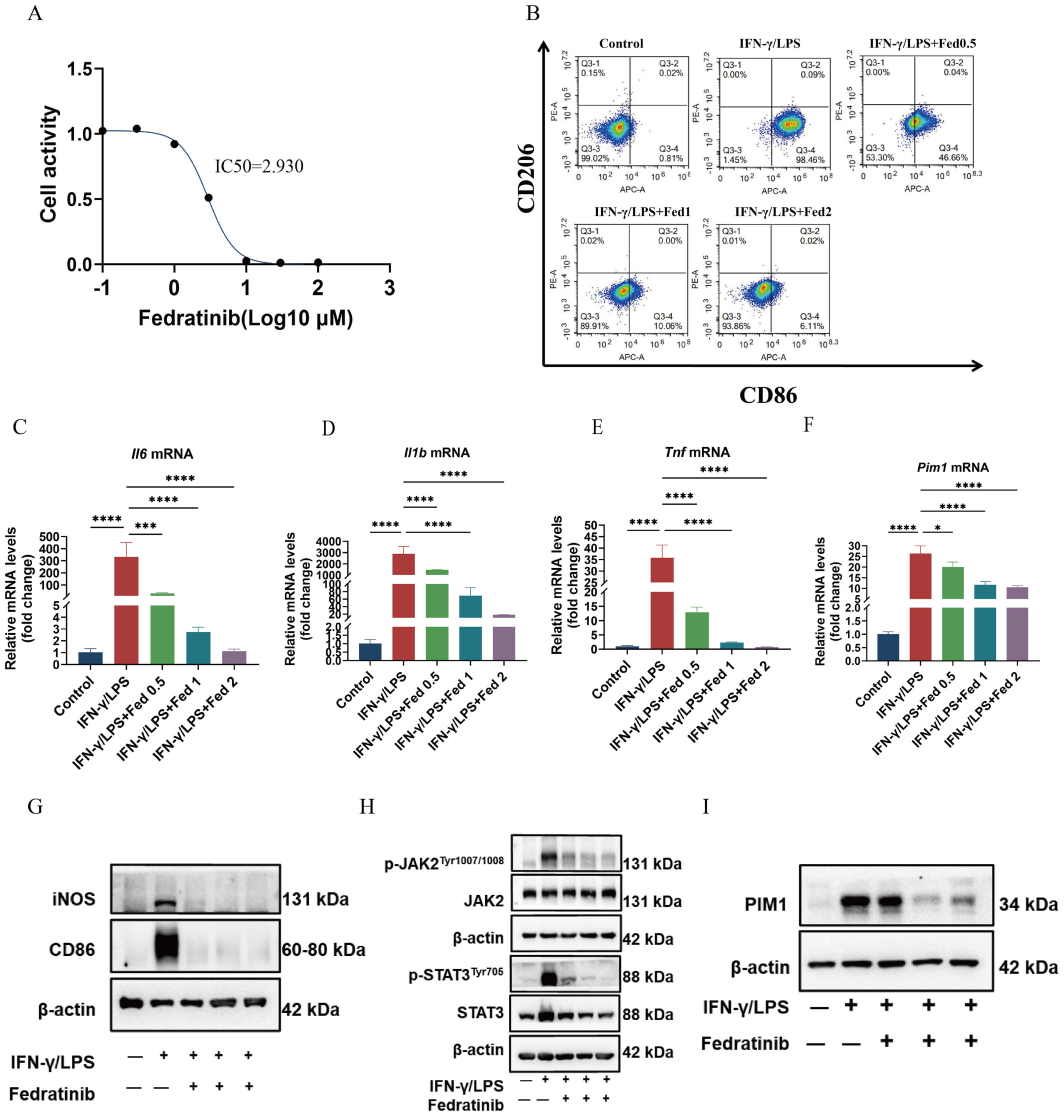
*In vitro* validation of Fedratinib inhibiting M1 polarization and the JAK2/STAT3/PIM1 axis. **(A)** Dose-response curve and IC50 determination of Fedratinib on RAW264.7 cells via CCK-8 assay. **(B)** Flow cytometry of CD86 (M1) and CD206 (M2) markers across Fedratinib concentrations. **(C–F)** RT-qPCR of pro-inflammatory genes (*Il6*, *Il1b*, *Tnf*) and *Pim1*. **(G)** Western Blot analysis of M1 polarization markers (iNOS, CD86). **(H)** Western Blot analysis of JAK2/STAT3 phosphorylation. **(I)** Western Blot analysis of PIM1 protein level.

Next, we conducted validation using RAW264.7 cells. Firstly, toxicity testing of Fedratinib on RAW264.7 cells revealed an IC_50_ value of 2.930 µM (**Figure 7A**). Flow cytometry confirmed that treatment with Fedratinib significantly reduced the proportion of IFN-γ/LPS-induced CD86+CD206- cells (**Figure 7B**). RT-qPCR results showed that Fedratinib significantly downregulated the mRNA expression levels of the pro-inflammatory genes *Il6*, *Il1b*, *Tnf* (**Figures 7C–E**) and *Pim1* (**Figure 7F**). Western blot analysis confirmed that Fedratinib effectively inhibited the expression of the polarizing proteins iNOS and CD86 (**Figure 7G**). Furthermore, whilst blocking the phosphorylation and activation of JAK2 (Tyr1007/1008) and its downstream target STAT3 (Tyr705) (**Figure 7H**), Fedratinib significantly reduced PIM1 protein levels (**Figure 7I**). These results indicate that Fedratinib, by disrupting the JAK2/STAT3/PIM1 signaling axis, effectively inhibits pro-inflammatory activation of macrophages, thereby reversing the trauma-induced pathological immune state.

## 4 Discussion

This study utilized multi-omics technologies to deconstruct the peripheral immune microenvironment of trauma and map it to ARDS. Our findings confirm that classical monocytes do not undergo homogeneous activation under traumatic stress. Instead, they differentiate into a highly pathogenic ISG15+ interferon-responsive subcluster and a potentially reparative MERTK+ subcluster. Subsequently, by analyzing ARDS patient alveolar macrophages, we validated the cross-tissue consistency of the peripheral-local immune signature centered on PIM1. The JAK2/STAT3/PIM1 axis exhibited synchronous expression in peripheral blood leukocytes of trauma patients, and TSMR analysis ultimately established elevated PIM1 expression as an independent causal driver of ARDS pathogenesis. These results identify the JAK2/STAT3/PIM1 axis as the core therapeutic targets and identify Fedratinib as a potential precision drug. In an M1 polarization cell model, we confirmed that Fedratinib effectively suppresses the M1 polarization phenotype by inhibiting the JAK2/STAT3/PIM1 signaling pathway.

Under homeostatic conditions, classical monocytes exist as a relatively homogeneous population primarily responsible for basal immunosurveillance (24). However, the single-cell pseudotime trajectory analysis in this study reveals that following severe trauma, this homeostatic population (cMo_S100A9+_VCAN+) does not undergo a uniform activation. Instead, it rapidly differentiates along two distinct trajectories. This divergence represents not only a remodeling at the transcriptomic level but also a fundamental polarization of functional phenotypes.

The cMo_ISG15+STAT1+ subcluster specifically overexpresses Type I interferon-stimulated genes (ISGs), including ISG15, IFITM1, IFI6, MX1, and the key transcription factor STAT1. Notably, ISG15 is not merely a transcriptional marker but also serves as a ubiquitin-like modifier protein (25). Under traumatic stress, intracellular ISG15 can modify newly synthesized proteins through covalent binding. While this process interferes with viral replication during viral infections, in the context of sterile inflammation, excessive ISGylation may lead to the accumulation of intracellular protein aggregates, thereby impairing autophagic flux (26–28). Furthermore, ISG15 can be secreted into the extracellular space, functioning as a cytokine-like signaling molecule. Research has demonstrated that ISG15 acts synergistically with IL-12 to induce IFN-γ secretion, thereby establishing a positive feedback loop that amplifies the inflammatory circuit (25, 29).

In fact, trauma causes extensive cell necrosis, leading to the leakage of mitochondrial DNA (mtDNA) into the cytoplasm or the systemic circulation (30). Since mtDNA is highly homologous to bacterial DNA, it can be recognized by the intracellular cGAS/STING pathway (31). Furthermore, under specific microenvironmental conditions, TNF-α signaling can independently induce ISG15 expression, establishing an autocrine loop (32, 33). This finding explains why patients with severe trauma exhibit an immune phenotype highly similar to that of patients with severe COVID-19 (34, 35). Further pathway enrichment and transcriptional regulatory analysis revealed significant activation of STAT3 or NF-κB signaling in this subcluster, providing a structural bridge between “trauma initiation–DAMPs–inflammatory storm.”

Our analytical workflow leverages an integrated multi-omics and computational drug-repositioning pipeline to bridge the gap between systemic immune dysregulation and local tissue pathology. This paradigm deeply aligns with modern systems-biology frameworks utilized to decode the highly heterogeneous landscape of critical care syndromic diseases; for example, network-based transcriptomic modeling and hub gene signatures have successfully uncovered novel therapeutic targets and shared driving pathways for sepsis and geriatric sepsis-induced ARDS (36). By echoing such network medicine strategies and intersecting circulating trauma monocyte signatures with local tissue multi-omics and genetic Mendelian randomization, our study expands the systems-biology repertoire for ARDS target discovery, establishing a robust translation path for Fedratinib.

To identify the most central and robust therapeutic targets, this study performed an intersection analysis between human single-cell data and mouse TLI model transcriptomes, identifying 166 overlapping genes. Among these, PIM1 was not only significantly upregulated but also demonstrated a causal association with ARDS pathogenesis via TSMR analysis. PIM1 is a direct downstream target gene of STAT3 and STAT5 and belongs to the serine/threonine kinase family (37). Its central role in traumatic immunopathology is manifested in three dimensions (1): Synergistic Inflammatory Amplification: PIM1 can directly phosphorylate the p65 subunit of NF-κB, enhancing its transcriptional activity, which promotes the continuous production of inflammatory cytokines such as IL-6, forming a STAT3/PIM1/NF-κB positive feedback loop (38) (2). Abrogation of Apoptotic Programs: PIM1 can phosphorylate the pro-apoptotic protein BAD, inactivating it and thereby significantly extending the lifespan of inflammatory cells (39) (3). Disruption of the Endothelial Barrier: Research indicates that the overexpression of PIM1 can lead to the degradation of junctional proteins in pulmonary microvascular endothelial cells, increasing vascular permeability and promoting the occurrence of pulmonary edema and ARDS (40). Furthermore, the TSMR analysis conducted in this study, which shares design principles with a randomized controlled trial by utilizing genetic randomization, provides robust genetic evidence supporting a potential causal contribution of elevated PIM1 expression to ARDS susceptibility, rather than it being a mere downstream consequence of the inflammatory process. This conclusion provides high-level preclinical evidence for pharmacological intervention targeting PIM1 or its upstream pathways.

The persistent presence of a pro-inflammatory myeloid phenotype that is difficult to resolve is a classic hallmark of severe lung injury. Recent advances in ALI models emphasize that the timely resolution of inflammation is a tightly coordinated process driven by precisely regulated immune cell axes; for example, studies have demonstrated that B cell-derived IL-10 plays a key role in promoting the resolution of LPS-induced ALI by inhibiting abnormal macrophage activation and limiting persistent leukocyte infiltration (41). Consistent with the paradigm of promoting resolution by targeting myeloid-driven excessive inflammation, this study demonstrates that inhibition of the JAK2/STAT3/PIM1 axis may offer a potent pharmacological approach to mitigating systemic inflammatory storms.

This study further used CMap predictions and validated them with the scRANK algorithm, revealing that the FDA-approved drug for myelofibrosis, Fedratinib (TG-101348), exhibits exceptionally high targeting sensitivity toward the cMo_ISG15+STAT1+ pro-inflammatory monocytes. Based on the mechanistic analysis above, this study proposes an intervention strategy targeting JAK2. The transcriptional program of the cMo_ISG15+STAT1+ subcluster is highly dependent on the continuous drive of the JAK/STAT pathway. Consequently, these cells are exquisitely sensitive to JAK2 blockade. Inhibiting JAK2 directly severs the transcriptional source of downstream pathogenic genes such as PIM1, effectively dismantling the initiation mechanism of the inflammatory storm (42, 43). Also, because the drug does not globally suppress all myeloid cells, the body retains a portion of its innate immune defenses, which are paramount for preventing secondary infections during the later stages of trauma recovery.

This study helps expand our understanding of trauma immunology at single-cell resolution. It provides a theoretical framework for shifting TLI treatment research from empirical anti-inflammation to mechanism-based cell subcluster reprogramming. Future research is still required to validate further the efficacy of this strategy in both *in vitro* and *in vivo* models. However, this study has certain limitations. First, being restricted to using peripheral blood samples, we were unable to directly capture the spatial distribution and real-time infiltration trajectories of the pathogenic cMo_ISG15+STAT1+ cell subcluster within human lung tissue *in situ*. It is critical to note that this proposed transcriptomic program primarily reflects a systemic inflammatory signature. While it is highly linked to local lung damage, direct spatial profiling is required to confirm whether these specific circulating cell subclusters match the exact phenotypes of lung-infiltrating myeloid subsets. Second, while we validated the intervention potential of the JAK2/STAT3/PIM1 signaling axis using Fedratinib, as an upstream inhibitor, it cannot completely isolate the independent pharmacological contribution of PIM1.

## 5 Conclusion

In conclusion, this study identifies a pathogenic cMo_ISG15+STAT1+ cell subcluster as a key driver of the post-traumatic inflammatory storm. The JAK2/STAT3/PIM1 axis is the core mechanism regulating the M1 polarization of cMo_ISG15+STAT1+ cells. Fedratinib, an FDA-approved drug, serves as a precision therapeutic strategy for treating TLI by targeting the JAK2/STAT3/PIM1 axis.

## Data Availability

The original contributions presented in the study are included in the article/**Supplementary Material**. Further inquiries can be directed to the corresponding author.
