# Supplementary material for: Targeting the ISG15+STAT1+ monocyte-driven inflammatory storm with Fedratinib in traumatic lung injury via the JAK2/STAT3/PIM1 axis
Source: Front Immunol. 2026 Jun 19;17:1843701. doi: 10.3389/fimmu.2026.1843701 (PMC13327986; doi:10.3389/fimmu.2026.1843701)
Supplement: Supplementary file 2 [file SupplementaryFile1.docx]

Targeting the ISG15+STAT1+ monocyte-driven inflammatory storm with Fedratinib in traumatic lung injury via the JAK2/STAT3/PIM1 axis

Kun Zhang^1^, Dan Li^1^, Le Gao^1^, and, Mingwei Chen^1*^

^1^ Department of Respiratory and Critical Care Medicine, First Affiliated Hospital of Xi'an Jiaotong University, Xi'an, 710061, Shaanxi, China

*** Correspondence:** Department of Respiratory and Critical Care Medicine, First Affiliated Hospital of Xi'an Jiaotong University, No. 277 Yanta West Road, Xi'an, 710061, Shaanxi Province, the People's Republic of China
Corresponding Author: Mingwei Chen

email: chenmw36@163.com.

# Supplementary Methods

# Cell Culture and Induction of M1 Polarization

The mouse macrophage cell line RAW 264.7 was purchased from Procell Life Science& Technology Co., Ltd. (Wuhan, China). Cells were cultured in high-glucose modified Dulbecco’s Modified Eagle’s Medium supplemented with 10% fetal bovine serum (Viva Cell, Shanghai, China). Cultures were maintained at 37°C in a 5% CO₂ incubator. To induce M1 polarization, cells were treated with a combination of IFN-γ (20 ng/mL) and LPS (100 ng/mL) when confluence reached 70–80%. According to the experimental design, cell samples were collected at specific time points for subsequent analysis.

# Detection of cell viability after Fedratinib intervention

The survival rates of RAW264.7 cells following Fedratinib treatment were determined using the CCK-8 assay. Cells in the logarithmic growth phase were seeded into 96-well plates (1 × 10⁴ cells/well, 100 μL medium/well) and cultured for 12 hours to promote cell adhesion. Subsequently, the cells were exposed to different concentrations of Fedratinib (0, 0.1, 0.3, 1, 3, 10, 30, and 100 μM) for 24 hours, with solvent and blank controls included; each condition comprised six replicate wells. Next, 10 μL of CCK-8 reagent was added to each well, and the plate was incubated at 37 °C in the dark for 1 hour. Absorbance was measured at 450 nm using a microplate reader. Cell viability was calculated by normalizing the blank-corrected absorbance values of each group to the absorbance value of the solvent control group.

# Flow Cytometry

Collect RAW 264.7 cells that have been processed as specified and pre-incubate them with anti-mouse CD16/32 antibody. Subsequently, perform surface staining with APC-labeled anti-CD86 antibody, then fix and permeabilize. Thereafter, perform intracellular staining with PE-labeled anti-CD206 antibody. Analyze the samples using a NovoCyte flow cytometer (ACEA Biosciences). Establish fluorescence compensation using a single-stain control, and process the data using NovoExpress software (ACEA Biosciences) to determine the proportions of CD86+ and CD206+ cell populations.

# Quantitative Real-Time PCR

Total RNA was extracted from RAW 264.7 cells using TRIzol reagent (Invitrogen, Carlsbad, CA, USA), and its purity was verified using a NanoDrop One spectrophotometer (Thermo Fisher Scientific, Waltham, MA, USA) based on an A260/A280 ratio of 1.8–2.0. 1 μg of total RNA was used for reverse transcription to synthesize cDNA using PrimeScript RT Master Mix (Takara Bio, Shiga, Japan). Perform PCR amplification on a CFX96 system (Bio-Rad, Hercules, CA, USA) using SYBR Green Master Mix (Jinsha, Beijing, China). Using Actb as an internal control, calculate the relative expression levels of the target genes (*Il1b*, *Il6*, *Pim1*, and *Tnf*) using the 2-ΔΔCt method.

# Antibodies and reagents

Primary antibodies: anti-PIM1 (3247T; Cell Signaling Technology, Danvers, MA, USA), anti-iNOS (18985-1-AP; Proteintech, Wuhan, China), anti-CD86 (19589T, Cell Signaling Technology, USA), anti-β-actin (AC026; ABclonal, Wuhan, China), anti-JAK2 (R24775, Zen-Bioscience, Chengdu, China), anti-p-JAK2Tyr1007/1008(R381556, Zen-Bioscience, Chengdu, China), anti-STAT3(10253-2-AP, Proteintech, Wuhan, China), anti-p-STAT3Tyr705(R381552, Zen-Bioscience, Chengdu, China), APC-conjugated anti-mouse CD86 antibody (105011, Biolegend), TruStain FcX™ PLUS (anti-mouse CD16/32) antibody (156603, Biolegend), PE-conjugated anti-mouse CD206 antibody (141705, Biolegend). Reagents: Lipopolysaccharide (LPS) (HY-D1056, MedChemExpress), recombinant mouse IFN-γ protein (HY-P7071, MedChemExpress).

# Western Blot

Total protein was extracted using RIPA lysis buffer containing protease and phosphatase inhibitors, and quantified using the BCA method. Equal volumes of protein were separated by SDS-PAGE and transferred to a PVDF membrane (Millipore, USA), which was then blocked with 5% skim milk. The membrane was then incubated sequentially with the primary antibody (overnight at 4°C) and the HRP-labeled secondary antibody (1 hour at room temperature). Target bands were detected using an ECL detection system (Tanon, Shanghai, China), and grayscale values were analyzed in ImageJ. Relative quantification was performed after normalization to an internal control.
